# Retraction: A Chaperonin Subunit with Unique Structures Is Essential for Folding of a Specific Substrate

**DOI:** 10.1371/journal.pbio.3001883

**Published:** 2022-11-04

**Authors:** Lianwei Peng, Yoichiro Fukao, Fumiyoshi Myouga, Reiko Motohashi, Kazuo Shinozaki, Toshiharu Shikanai

Following the publication of this article and the Expression of Concern notice [1,2], the Kyoto University conducted an investigation into the concerns raised and found that some of the errors with figures presented in this article were the result of fraudulent image manipulation [3]. In light of the institutional findings of misconduct, the corresponding author requested the retraction of this article.

All authors agreed with the retraction. LP, YF, FM, RM, and TS stand by the article’s findings. LS and TS apologise for the issues with the published article.
