# Correction: Retraction: A Chaperonin Subunit with Unique Structures Is Essential for Folding of a Specific Substrate

**DOI:** 10.1371/journal.pbio.3001925

**Published:** 2022-12-02

**Authors:** The PLOS Biology Editors

There is an error in the final sentence of the Notice of Retraction [1]. The correct sentence is: LP and TS apologize for the issues with the published article.

The publisher apologizes for the error.
